# Dehydrogenase reductase 9 (SDR9C4) and related homologs recognize a broad spectrum of lipid mediator oxylipins as substrates

**DOI:** 10.1016/j.jbc.2021.101527

**Published:** 2021-12-22

**Authors:** Olga V. Belyaeva, Samuel E. Wirth, William E. Boeglin, Suman Karki, Kelli R. Goggans, Stacy G. Wendell, Kirill M. Popov, Alan R. Brash, Natalia Y. Kedishvili

**Affiliations:** 1 Department of Biochemistry and Molecular Genetics, University of Alabama at Birmingham, Birmingham, Alabama, USA; 2 Department of Pharmacology, Vanderbilt University, Nashville, Tennessee, USA; 3 Department of Pharmacology and Chemical Biology, School of Medicine, University of Pittsburgh, Pittsburgh, Pennsylvania, USA

**Keywords:** eicosanoids, docosanoids, dehydrogenase, polyunsaturated lipids, retinol, steroids, 14(*S*)-HDoHE, 14(*S*)- hydroxy-4Z,7Z,10Z,12E,16Z,19Z-docosahexaenoic acid, 12(*S*)-HETE, 12(*S*)-hydroxy-(5Z,8Z,10E,14Z)-eicosatetraenoic acid, 15(*S*)-HETE, 15(*S*)-hydroxy-(5Z,8Z,11Z,13E)-eicosatetraenoic acid, 9(*S*)-HODE, 9S-hydroxy-10E,12Z-octadecadienoic acid, 13(*S*)-HODE, 13(*S*)- hydroxy-9*Z*,11*E*-octadecadienoic acid, LTB_4_, leukotriene B_4_, LXA_4_, lipoxin A_4_, RvD1, resolvin D1

## Abstract

Bioactive oxylipins play multiple roles during inflammation and in the immune response, with termination of their actions partly dependent on the activity of yet-to-be characterized dehydrogenases. Here, we report that human microsomal dehydrogenase reductase 9 (DHRS9, also known as SDR9C4 of the short-chain dehydrogenase/reductase (SDR) superfamily) exhibits a robust oxidative activity toward oxylipins with hydroxyl groups located at carbons C9 and C13 of octadecanoids, C12 and C15 carbons of eicosanoids, and C14 carbon of docosanoids. DHRS9/SDR9C4 is also active toward lipid inflammatory mediator dihydroxylated Leukotriene B_4_ and proresolving mediators such as tri-hydroxylated Resolvin D1 and Lipoxin A_4_, although notably, with lack of activity on the 15-hydroxyl of prostaglandins. We also found that the SDR enzymes phylogenetically related to DHRS9, *i.e.*, human SDR9C8 (or retinol dehydrogenase 16), the rat SDR9C family member known as retinol dehydrogenase 7, and the mouse ortholog of human DHRS9 display similar activity toward oxylipin substrates. Mice deficient in DHRS9 protein are viable, fertile, and display no apparent phenotype under normal conditions. However, the oxidative activity of microsomal membranes from the skin, lung, and trachea of *Dhrs9*^*−/−*^ mice toward 1 μM Leukotriene B_4_ is 1.7- to 6-fold lower than that of microsomes from wild-type littermates. In addition, the oxidative activity toward 1 μM Resolvin D1 is reduced by about 2.5-fold with DHRS9-null microsomes from the skin and trachea. These results strongly suggest that DHRS9 might play an important role in the metabolism of a wide range of bioactive oxylipins *in vivo*.

The short-chain dehydrogenase/reductase (SDR) superfamily of proteins is comprised of over 47,000 members that are found in all forms of life (1, 2, 3). Members are assigned on the basis of SDR signature features, including the TGX_3_GXG motif of the nucleotide-binding region and the catalytically active tetrad N-S-Y-K, which constitutes the active site. Typically, these proteins function as dimeric or tetrameric NAD(P)(H)-dependent oxidoreductases with a subunit molecular mass of 25 to 35 kDa. SDR enzymes are found in the cytoplasm, mitochondria, nuclei, peroxisomes, and endoplasmic reticulum where they participate in the metabolism of sugars, steroids, prostaglandins, retinoids, aliphatic alcohols, and xenobiotics.

DHRS9 belongs to SDR9C, the largest subfamily of SDRs with many as eight human members and at least 13 mouse homologs. The additional mouse homologs likely appeared as a result of multiple rounds of lineage-specific gene duplications (Fig. 1) (3, 4). The two founding members were named retinol dehydrogenases (RDH), based on the initial assays that demonstrated their activities toward 11-*cis*-retinol (human RDH5), (5) and all-*trans*-retinol (rat RDH7) (6). However, later analysis revealed that both RDH5 and RDH7 displayed activities toward 3α-hydroxysteroids (7, 8). In the case of rat RDH7, its 3α-hydroxysteroid dehydrogenase activity significantly exceeded that with all-*trans*-retinol. Similarly, human homologs of rat RDH7, *i.e.*, human RDH16 (SDR9C8), which shares 72% protein sequence identity with rat RDH7, and human 17β-hydroxysteroid dehydrogenase 6 (HSD17B6/SDR9C6) with 66% sequence identity (Fig. 1) to rat RDH7 were shown to oxidize the hydroxyl groups on steroid molecules (9, 10). Furthermore, like human RDH5 and rat RDH7, human RDH16 and HSD17B6 were also capable of oxidizing retinoid substrates, suggesting that active sites of these enzymes can accommodate structurally unrelated substrates (names of these SDRs assigned by different researchers who independently cloned the corresponding genes are listed in Table S1).

**Figure 1:**
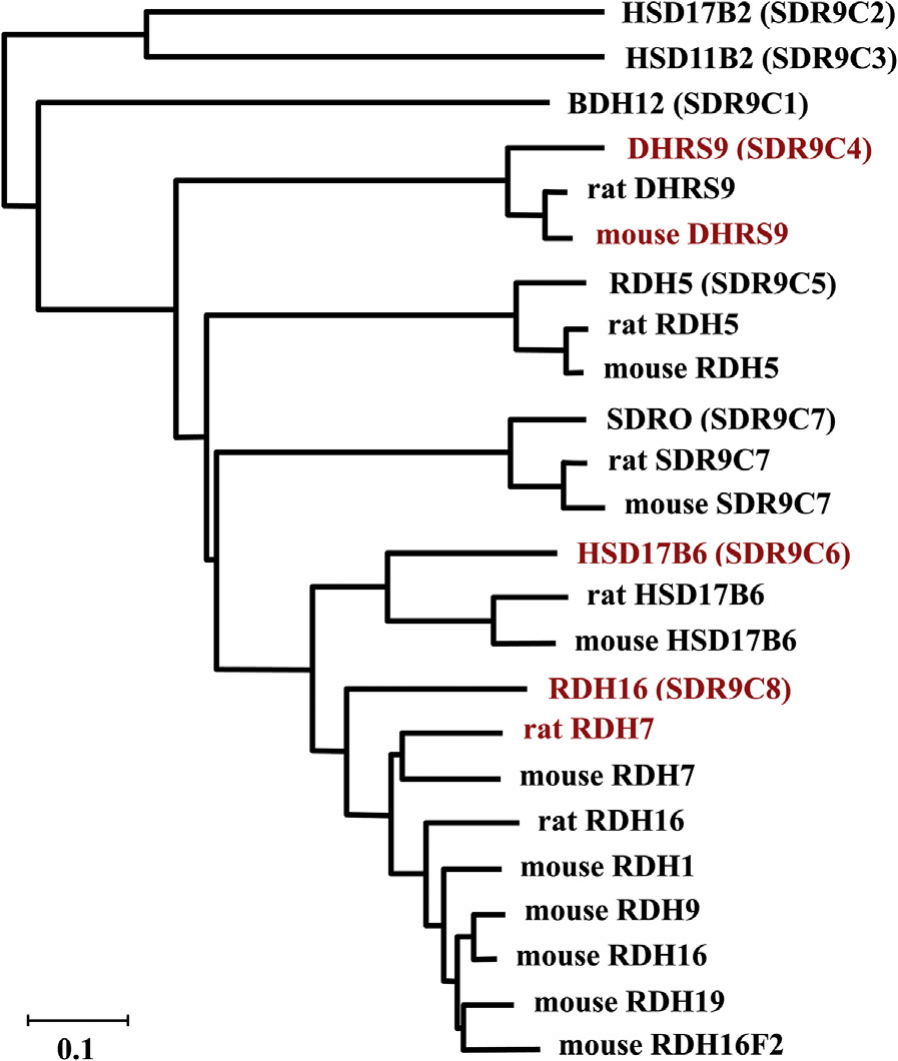
**Phylogenetic tree of human and rodent members of the SDR9C family of proteins.** The phylogeny of SDR9C family was inferred using the Neighbor-Joining method (52). The optimal tree is shown. The tree is drawn to scale, with branch lengths in the same units as those of the evolutionary distances used to infer the phylogenetic tree. The evolutionary distances were computed using the Poisson correction method and are in the units of the number of amino acid substitutions per site. This analysis involved 24 amino acid sequences. There were a total of 489 positions in the final dataset. Analysis was conducted in MEGA X (53). Proteins investigated in this study are highlighted in *red font*.

Although *in vitro* human RDH5 (SDR9C5) displays dual substrate specificity toward 11-*cis*-retinol and 3α-hydroxysteroids, its *in vivo* role in the oxidation of 11-*cis*-retinol to 11-*cis*-retinaldehyde in the visual cycle has been supported by genetic linkage evidence (11) and gene knockout analysis in a mouse model [reviewed in ref. (12). In addition to the established role of RDH5 in 11-*cis*-retinol metabolism, a recent study uncovered the role of SDR9C7, previously known as SDR-orphan (SDR-O), in dehydrogenation of acylceramides for skin barrier formation (13, 14). This finding further emphasized the idea that members of SDR9C family can accommodate various structurally unrelated substrates.

DHRS9 (SDR9C4) is one of the least understood proteins of the 9C family. Originally identified based on its sequence similarity to RDHs (∼50%), DHRS9 was initially tested with retinoids and hydroxysteroids as candidate substrates. Data from this laboratory showed that DHRS9 catalyzed the oxidation of 3α-hydroxyl group on androgenic 3α-hydroxysteroids and neurosteroids (15). Others reported that DHRS9 exhibited a retinol dehydrogenase activity (16, 17, 18). Accordingly, DHRS9 has been known under several different names (see Table S1), and it is currently believed that DHRS9 functions as a retinol dehydrogenase (19) or as a neurosteroid dehydrogenase (20). However, as indicated by our previous studies, the activity of DHRS9 toward neurosteroid allopregnanolone or androsterone was significantly lower than that of either HSD17B6 or RDH16 (21), and in our hands, the activity of DHRS9 toward all-*trans*-retinol was largely negligible. Consistent with our kinetic measurements, it was shown that unlike the well-established retinol dehydrogenase RDH10 of the SDR16C family (22), DHRS9 does not contribute to the oxidation of retinol in the pathway of retinoic acid biosynthesis when overexpressed in organotypic skin rafts (23). Thus, taking into account that DHRS9 displayed fairly modest activity with steroid substrates and little if any activity with retinoid substrates, these findings suggested that DHRS9 might naturally utilize substrates other than steroids and/or retinoids.

Considering that human DHRS9 mRNA has a broad tissue distribution pattern (15), and the expression of DHRS9 is frequently altered during tissue inflammation and/or carcinogenesis as well as in metabolic disorders and obesity (24, 25, 26, 27, 28, 29, 30, 31, 32, 33), we reinvestigated the substrate specificity of human and rodent DHRS9. The results of this study suggest an evolutionarily conserved role for DHRS9 in the oxidative metabolism of a broad range of bioactive oxylipins.

## Results

### Substrate specificity of SDR9C family member DHRS9 (SDR9C4)

Human DHRS9 (SDR9C4) was expressed in Sf9 cells using the baculovirus expression system, and the microsomes were isolated for kinetic analysis. In addition to DHRS9, other members of SDR9C family with reported retinoid and hydroxysteroid activities, *i.e.*, human RDH16 (9), human HSD17B6 (10), and rat RDH7 (8) were assayed with the same set of previously untested substrates.

**Figure 2:**
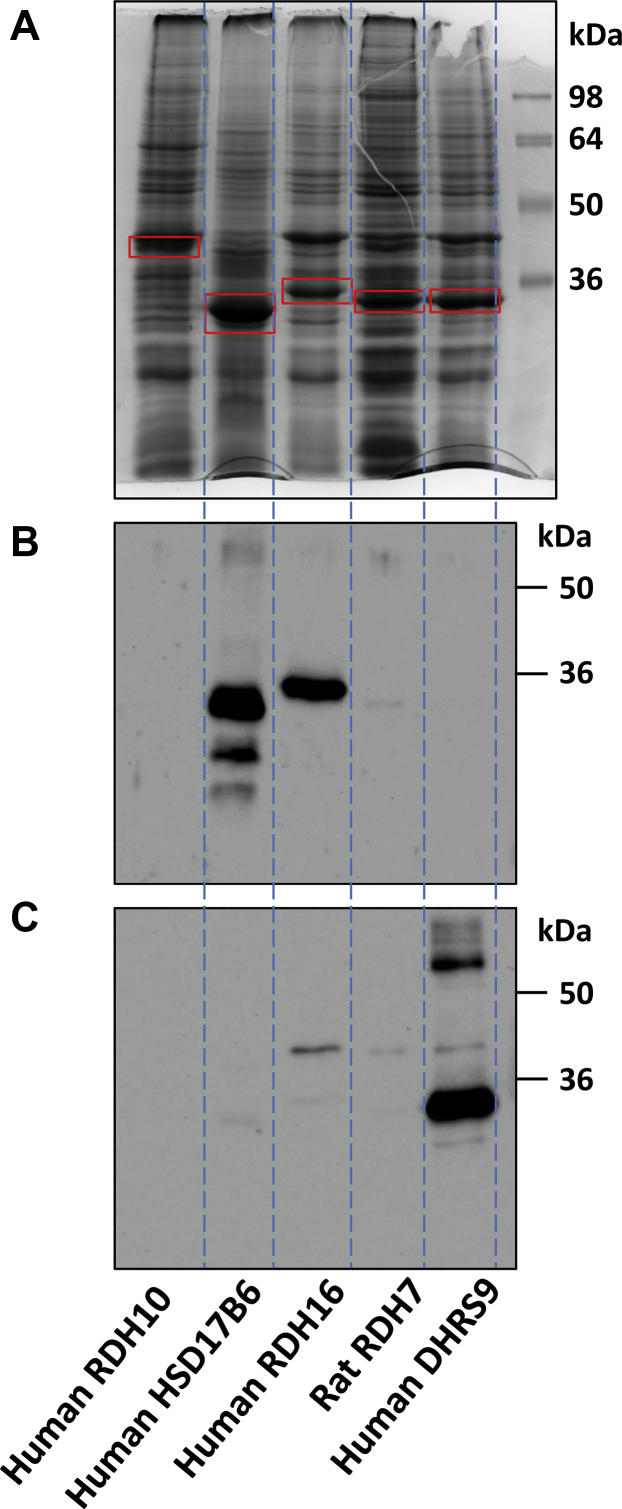
**Expression of SDR9C proteins in Sf9 microsomes.***A*, Coomassie Blue-stained SDS-PAGE gel. Fifty micrograms of Sf9 microsomes containing different recombinant proteins were loaded into each lane. RDH10 microsomes were loaded as a negative control. The positions of each recombinant protein are indicated by the *red boxes*. Based on scanning densitometry, the protein content of each SDR in microsomal preparations relative to human RDH16, which was set as 1, was as follows: 3.15-fold higher for human HSD17B6, 1.63-fold higher for rat RDH7, and 1.6-fold higher for human DHRS9. *B*, Western blot analysis of the same set of microsomal preparations (0.5 μg/lane) using human RDH16 (RoDH4) antibodies (N-terminal M17, 1:5000) (9). Note that the antibodies detect human HSD17B6 protein and also weakly react with rat RDH7. *C*, Western blot analysis of the same set of microsomal preparations (0.5 μg/lane) using DHRS9 antibodies (Abclonal, 1:2000).

The relative levels of each protein in Sf9 microsomes were assessed using Coomassie Blue stained SDS-PAGE gels (Fig. 2*A*). The identities of each SDR were verified by Western blotting (Fig. 2, *B* and *C*) and quantified by scanning densitometry. Although the microsomal preparations contained somewhat different protein amounts of individual SDRs, considering that protein levels of RDH16, DHRS9, and RDH7 were fairly close, some ideas about their relative catalytic efficiencies and substrate specificities can be drawn from the kinetic parameters.

As summarized in Table 1, DHRS9 displayed a very robust activity toward hydroxylated oxylipins (structures of substrates tested are shown in Fig. S1). Of significance regarding substrate specificity, within the arachidonic acid series of HETEs, we were unable to detect any activity with 5(*S*)-HETE, whereas both 12(*S*)-HETE and 15(*S*)-HETE were well oxidized, suggesting that DHRS9 preferentially oxidizes secondary alcohol groups located along the CH_3_-terminal tail of the carbon chain. This was supported by the relative oxidation rates of the two hydroxy-C18:2ω6 oxylipins: both were effective substrates, with a fivefold higher rate for 13(*S*)-HODE compared with 9(*S*)-HODE. In line with this specificity, 14(*S*)-HDoHE of the C22:6ω3 series was also a good substrate.

**Table 1:** Substrate specificity of human DHRS9 (SDR9C4)

Substrate	*K*_m_, μM	*V*_max_^a^, pmol × min^−1^	*V*_max_/*K*_m_ pmol/(min × μM)
12(*S*)-HETE	0.43 ± 0.16	220 ± 33	512
15(*S*)-HETE	0.36 ± 0.18	140 ± 21	390
9(*S*)-HODE	0.57 ± 0.25	24 ± 3	42
13(*S*)-HODE	0.46 ± 0.19	110 ± 14	240
14(*S*)-HDoHE	0.17 ± 0.06	80 ± 8	471
LTB_4_	0.76 ± 0.44	170 ± 43	224
LXA_4_	4.6 ± 2.7	230 ± 76	50
RvD1	1.6 ± 0.5	85 ± 12	53
All-*trans*-retinol	ND	0.32^b^	-
Androsterone	ND	6.8^c^	-

Abbreviation: ND, not determined.

a To facilitate the comparison of catalytic efficiency of DHRS9 toward various oxylipin substrates, its *V*_max_ values were calculated based on the activity in samples containing 1 μg of total microsomal protein.

b The reaction rate was determined using 10 μM all-*trans*-retinol and 1 mM NAD^+^ with 5 μg of DHRS9 Sf9 microsomes.

c The reaction rate was determined using 20 μM androsterone and 1 mM NAD^+^ with 5 μg of DHRS9 Sf9 microsomes.

With polyhydroxylated substrates (Fig. S1), DHRS9 demonstrated the greatest *V*_max_ value toward LXA_4_, followed by LTB_4_ and RvD1 in the order: LXA_4_ > LTB_4_ >> RvD1. However, its *K*_m_ value for LXA_4_ was sixfold greater than that for LTB_4_ (Table 1). Consequently, the catalytic efficiency (*V*_max_/*K*_m_) of DHRS9 for LTB_4_ exceeded that of LXA_4_ and RvD1 by a factor of 4.5 and 4.2, respectively, suggesting that among LXA_4_, LTB_4_, and RvD1, LTB_4_ might be the preferred substrate of human DHRS9.

In designating DHRS9-catalyzed oxidation to classes of potential substrates, it is notable that the activity with the top oxylipin substrates greatly exceeded the rates with androsterone and all-*trans*-retinol, by 20-fold and 500-fold, respectively, compared with 12(*S*)-HETE (Table 1 and Fig. S2). These observations strongly suggest that hydroxylated oxylipins are the preferred, natural substrates of DHRS9.

**Figure 3:**
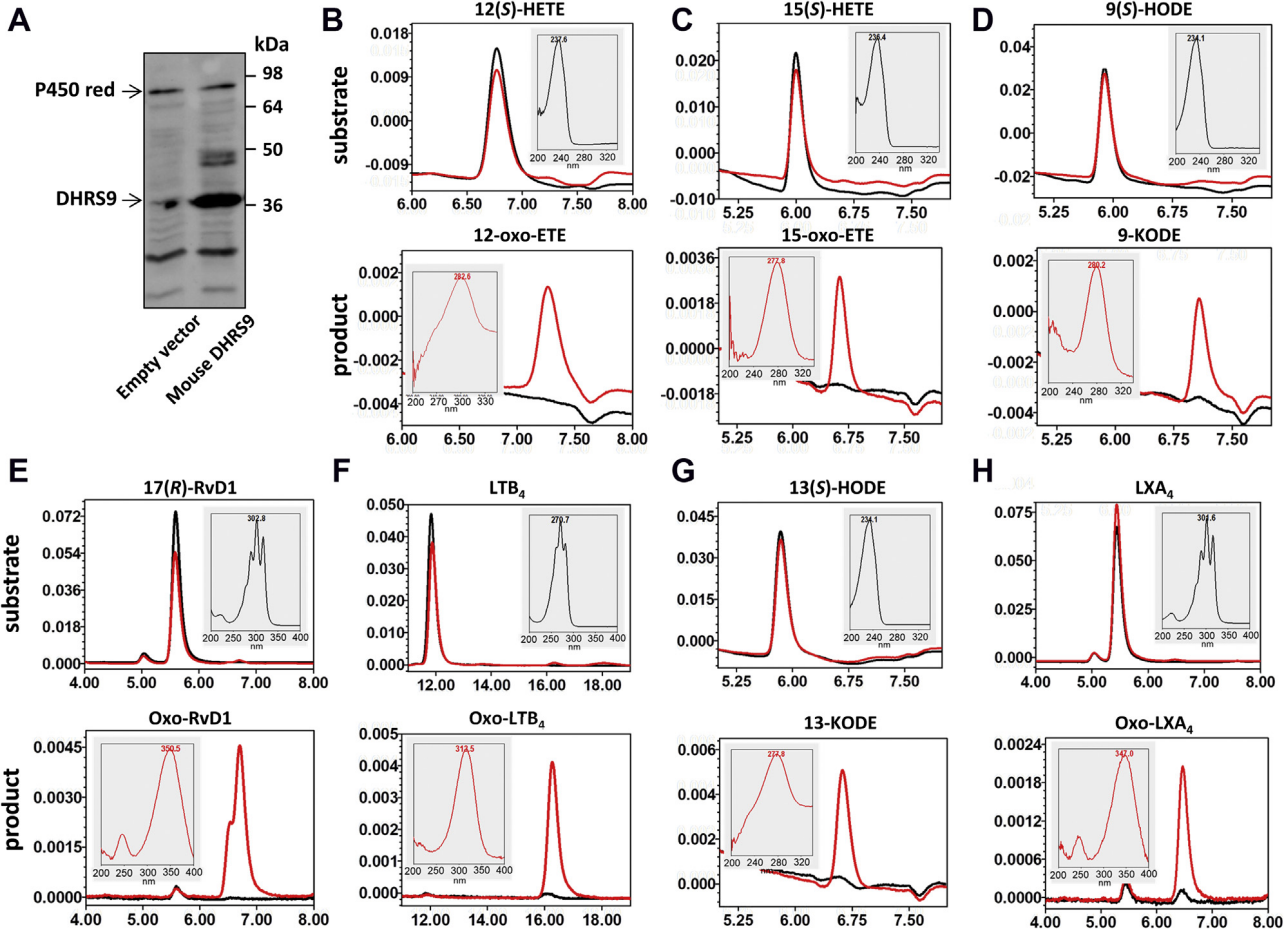
**Characterization of mouse DHRS9 activities towards oxylipins.***A*, Western blot analysis of mouse DHRS9 expression in HEK 293 cells. HEK 293 cells were transiently transfected with mouse DHRS9 expression construct. HEK293 microsomes (30 μg) containing DHRS9 protein were analyzed by Western blotting using DHRS9 antibodies (Abclonal, 1:2000). Immunodetection with cytochrome P450 reductase antibodies (#13513 Abcam, 1:1000) was used for a gel loading control. *B*–*H*, activities of mouse DHRS9. Microsomes (5 μg) isolated from HEK293 cells transfected with either empty vector (*black traces*) or mouse *Dhrs9* expression construct (*red traces*) were incubated with substrates (1 μM each) as indicated. The reaction products were extracted and analyzed as described under Experimental procedures. *Top panels*—chromatograms extracted at the λ_max_ of the substrate, *bottom panels*—at λ_max_ of the product. Note the appearance of the oxidized products in the presence of DHRS9. *Insets*, spectra for substrates (*black*) and products (*red*).

Comparable studies with the mouse enzyme used microsomes from DHRS9-expressing HEK293 cells. Qualitatively, the activity on oxylipins was similar to the human enzyme (Fig. 3 and Table 2). Among the monohydroxylated oxylipins, the highest rate of conversion was observed with 12(*S*)-HETE. 15(*S*)-HETE, 9(*S*)-HODE, and 13(*S*)-HODE were oxidized at approximately similar rates that were about twofold lower than with 12(*S*)-HETE (Table 2). With polyhydroxylated substrates, mouse DHRS9 demonstrated the highest oxidation rate toward 17(*R*)-RvD1, followed by LTB_4_. The reaction rate was about threefold lower with 1 μM LXA_4_ as substrate compared with 1 μM RvD1, and no activity was detected toward LXB_4_. On account of the relatively low expression level in HEK293 cells, a means to quantify mouse DHRS9 protein levels was lacking, obviating direct comparison of rates with human DHRS9, although the substrate specificities were an excellent match.

**Table 2:** Activity of mouse DHRS9 toward oxylipins

Substrate	Product	Rate, nmol × min^−1^ × mg^−1^ of microsomal protein
12(*S*)-HETE	12-oxo-ETE	1.9 ± 0.2
15(*S*)-HETE	15-oxo-ETE	1.1 ± 0.1
9(*S*)-HODE	9-KODE	0.82 ± 0.05
13(*S*)-HODE	13-KODE	0.70 ± 0.03
RvD1	Oxo-RvD1	1.2 ± 0.1
17(*R*)-RvD1	Oxo-RvD1	1.3 ± 0.1
LXA_4_	Oxo-LXA_4_	0.30 ± 0.02
LXB_4_	Oxo-LTB_4_	0.0
LTB_4_	Oxo-LTB_4_	0.88 ± 0.04

Microsomes (5 μg) isolated from HEK293 cells transfected with mouse DHRS9 expression construct were incubated with substrates (1 μM each) as indicated. The reaction products were extracted and analyzed as described under Experimental procedures. The values represent the reaction rates after subtracting the rates obtained with microsomes from HEK293 cells transfected with empty vector.

### Substrate specificity of RDH16 (SDR9C8)

Similar analysis carried out with another member of the SDR9C family human RDH16 (SDR9C8) yielded significant differences. Although 15(*S*)-HETE and the two C18:2 HODEs were oxidized by RDH16 (Table 3), the enzyme displayed 1 to 2 orders of magnitude lower rates compared with DHRS9, even after accounting for the 1.6-fold lower level of RDH16 protein expression in Sf9 microsomes (see Fig. 2*A*); furthermore, there was negligible activity with 12(*S*)-HETE. RDH16 also exhibited marginal activities with LTB_4_, LXA_4_, RvD1, and all-*trans*-retinol, making it highly unlikely that RDH16 can successfully compete *in vivo* with DHRS9 for these oxylipins, or with RDH10, the proven retinol dehydrogenase (22), for all-*trans*-retinol.

**Table 3:** Substrate specificity of human RDH16 (SDR9C8)

Substrate	*K*_m_, μM	*V*_max_^#^, pmol × min^−1^	*V*_max_/*K*_m_ pmol/(min × μM)
12(*S*)-HETE	2.01 ± 0.64	2.15 ± 0.24	1
15(*S*)-HETE	1.39 ± 0.56	20.43 ± 3.29	15
9(*S*)-HODE	1.76 ± 0.59	16.72 ± 1.88	10
13(*S*)-HODE	0.83 ± 0.36	7.70 ± 1.16	9
LTB_4_	ND	1.22^a^	-
LXA_4_	ND	1.1^a^	-
RvD1	ND	0.21^a^	-
All-*trans*-retinol	ND	0.97^d^	0.1^c^
Androsterone	ND	5.2^b^	24^c^

Abbreviation: ND, not determined.

^#^To facilitate the comparison of catalytic efficiency of RDH16 toward various oxylipin substrates, its *V*_max_ values were calculated based on the activity in samples containing 1 μg of total microsomal protein.

a The reaction rate was determined using 4 μM substrate and 20 μg of Sf9 microsomes containing the respective enzyme.

b The reaction rate was determined using 20 μM androsterone and 1 mM NAD^+^ with 5 μg of RDH16 Sf9 microsomes.

c Data from ref. (34).

d The reaction rate was determined using 10 μM all-*trans*-retinol and 1 mM NAD^+^ with 5 μg of RDH16 Sf9 microsomes.

In fact, the *V*_max_/*K*_m_ values of RDH16 for monohydroxylated oxylipins were lower than its *V*_max_/*K*_m_ value for androsterone (Table 3 and Fig. S2) that we reported previously (34). Taken together, these observations suggest that it is highly unlikely that RDH16 can directly compete for monohydroxylated oxylipins with DHRS9 or for androsterone with other more specialized sterol dehydrogenases. It is still possible that RDH16 might occupy some specialized niche where it can metabolize oxylipins and/or steroids without directly competing with other better suited enzymes. Alternatively, it might indicate that natural substrates of RDH16 remain to be identified.

### Substrate specificity of human HSD17B6 (SDR9C6)

Kinetic analysis of human HSD17B6 revealed marginal activities with the majority of oxylipin substrates as well as with all-*trans*-retinol (Table 4). Therefore, it is highly unlikely that HSD17B6 can compete with DHRS9 for any biologically active oxylipin substrates. It is also unlikely that HSD17B6 can compete with specialized retinol dehydrogenases, like RDH10, for all-*trans*-retinol. In fact, these outcomes are not particularly surprising because our earlier study (10) had demonstrated that HSD17B6 acts as a robust sterol dehydrogenase showing high catalytic efficiency for androsterone (see Table 3 and Fig. S2).

**Table 4:** Substrate specificity of human HSD17B6 (SDR9C6)

Substrate	*K*_m_, μM	*V*_max_^#^, pmol × min^−1^	*V*_max_/*K*_m_ pmol/(min × μM)
12(*S*)-HETE	0.87 ± 0.35	7.96 ± 0.89	9
15(*S*)-HETE	1.78 ± 0.42	2.88 ± 0.23	1.6
9(*S*)-HODE	0.64 ± 0.28	0.40 ± 0.05	0.6
13(*S*)-HODE	0.33 ± 0.24	0.9 ± 0.2	2.7
LTB_4_	ND	0.13^a^	-
LXA_4_	ND	0.27^a^	-
RvD1	ND	1.84^a^	-
All-*trans*-retinol	ND	3.6^d^	0.38^c^
Androsterone	ND	29.5^b^	150^c^

Abbreviation: ND, not determined.

^#^To facilitate the comparison of catalytic efficiency of HSD17B6 toward various oxylipin substrates, its *V*_max_ values were calculated based on the activity in samples containing 1 μg of total microsomal protein.

a The reaction rate was determined using 4 μM substrate and 20 μg of Sf9 microsomes containing the respective enzyme.

b The reaction rate was determined using 20 μM androsterone and 1 mM NAD^+^ with 5 μg of HSD17B6 Sf9 microsomes.

c Data from ref. (10).

d The reaction rate was determined using 10 μM all-*trans*-retinol and 1 mM NAD^+^ with 5 μg of HSD17B6 Sf9 microsomes.

### Substrate specificity of rat RDH7

Rat RDH7 is a homolog of human RDH16 (Fig. 1). This enzyme displayed only marginal activities with polyhydroxylated oxylipin substrates (Table 5). However, similar to human RDH16, rat RDH7 could oxidize some of monohydroxylated oxylipins such as 9(*S*)-HODE, 15(*S*)-HETE, and 12(*S*)-HETE. Furthermore, unlike human RDH16, which had *K*_m_ values in the range of 1.5 to 2 μM, rat RDH7 displayed low, submicromolar *K*_m_ values for 9(*S*)-HODE and 15(*S*)-HETE (0.1–0.2 μM), making it fairly specific for these substrates.

**Table 5:** Substrate specificity of rat RDH7

Substrate	*K*_m_, μM	*V*_max_^#^, pmol × min^−1^	*V*_max_/*K*_m_ pmol/(min × μM)
12(*S*)-HETE	0.76 ± 0.42	3.35 ± 0.53	4.4
15(*S*)-HETE	0.10 ± 0.09	13.7 ± 2.7	137
9(*S*)-HODE	0.18 ± 0.09	15.1 ± 2.2	84
LTB_4_	ND	0.23^a^	-
LXA_4_	ND	0.61^a^	-
RvD1	ND	0.33^a^	-
All-*trans*-retinol	ND	4.4^c^	-
Androsterone	ND	34.4^b^	-

Abbreviation: ND, not determined.

^#^To facilitate the comparison of catalytic efficiency of rat RDH7 toward various oxylipin substrates, its *V*max values were calculated based on the activity in samples containing 1 μg of total microsomal protein.

a The reaction rate was determined using 4 μM substrate and 20 μg of Sf9 microsomes containing the respective enzyme.

b The reaction rate was determined using 20 μM androsterone and 1 mM NAD^+^ with 5 μg of rat RoDH1 Sf9 microsomes.

c The reaction rate was determined using 10 μM all-*trans*-retinol and 1 mM NAD+ with 5 μg of rat RDH7 Sf9 microsomes.

In addition to 9(*S*)-HODE and 15(*S*)-HETE, rat RDH7 showed a very high activity toward androsterone (Fig. S2), suggesting that, *in vivo*, in addition to the metabolism of oxylipins, rat RDH7 might also contribute to the metabolism of sterols. Lastly, RDH7 activity toward all-*trans*-retinol was rather modest (Table 5), making it fairly unlikely that it contributes to the retinoid metabolism *in vivo*.

In addition to the four members of the SDR9C family, we analyzed the ability of several other SDRs from different families to utilize oxylipin substrates. We were unable to detect an appreciable activity for these substrates with retinoid-active SDRs such as human RDH10 (SDR16C4) (35) and RDH5 (SDR9C5) (5) or steroid-active SDR HSD11B1 (SDR26C1) (data not shown).

### Generation of *Dhrs9*^*−/−*^ mice

**Figure 4:**
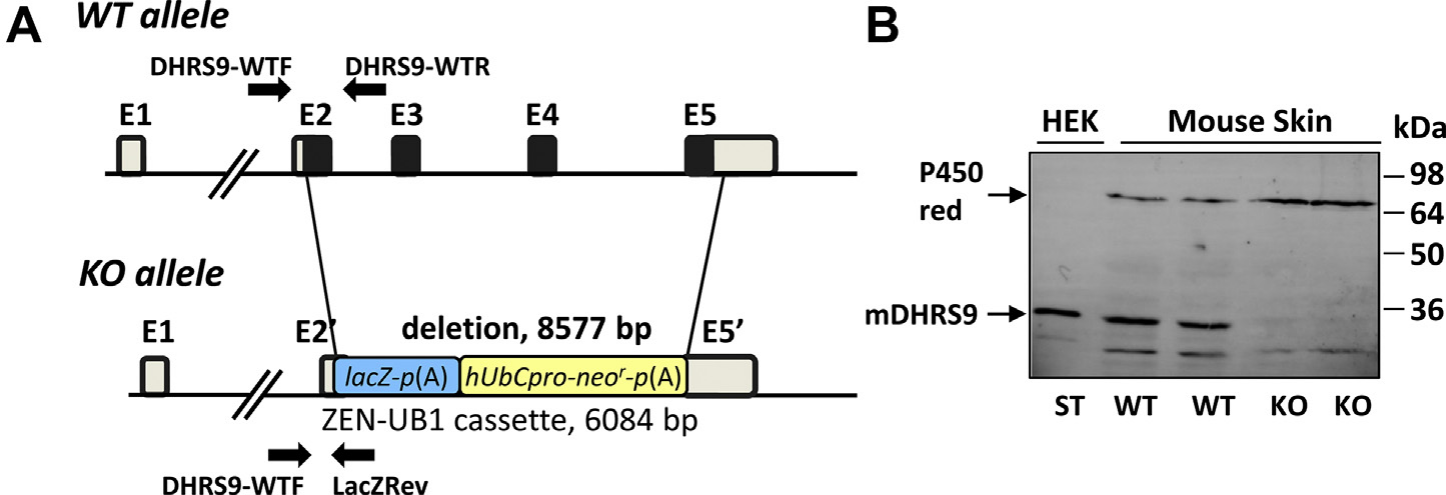
**Generation of *Dhrs9***^***−/−***^**mice.***A*, diagram of *Dhrs9*tm1(KOMP)Vlcg knockout allele. Velocigene KOMP Definitive Null Allele Design #13240 replaces 8577 bp of mouse chromosome 2 with Velocigene ZEN-UB1 cassette encoding promoterless LacZ reporter and human ubiquitin C promoter-driver neomycin selection marker. The generated allele lacks the complete protein-coding sequence of *Dhrs9* gene and expresses LacZ under the control of endogenous *Dhrs9* promoter. Coding exons (E2–E5) are shown as *black rectangles*, noncoding E1 and portions of E2 and E5—as *gray rectangles*. Positions of genotyping primers are indicated by the *black arrows*. *B*, validation of *Dhrs9* gene knockout by Western blot analysis. Microsomes (80 μg) isolated from skin of two wild-type (WT) and two *Dhrs9* KO mice were separated by SDS-PAGE and transferred to PVDF Immobilon–P membrane. The membrane was incubated with rabbit polyclonal DHRS9 antibodies (1:250 dilution) obtained from Dr Helen Everts at Texas Woman's University. Microsomes (20 μg) isolated from HEK 293 cells (HEK) transfected with mouse DHRS9 (mDHRS9) expression construct were used as a standard (St. DHRS9). Immunodetection with cytochrome p450 ab 13513 (Abcam, 1:1000) was used for a gel loading control.

To establish whether the native endogenously expressed DHRS9 contributes to the metabolism of oxylipins, we generated a mouse model with targeted knockout of *Dhrs9* gene. *Dhrs9*^*−/−*^ mouse strain has been generated by the UAB Transgenic Models Core *via* blastocyst injections of mouse embryonic stem cells carrying targeted allele of *Dhrs9* gene obtained from NIH Knockout Mouse Project (KOMP) (Fig. 4*A*). The absence of DHRS9 protein in the skin microsomes from *Dhrs9*^*−/−*^ mice was confirmed by Western blot analysis (Fig. 4*B*). *Dhrs9*^*−/−*^ mice were viable and fertile and did not display an obvious phenotype under normal conditions.

### LTB_4_ and RvD1 dehydrogenase activities are reduced in *Dhrs9*^*−/−*^ mouse tissues

**Figure 5:**
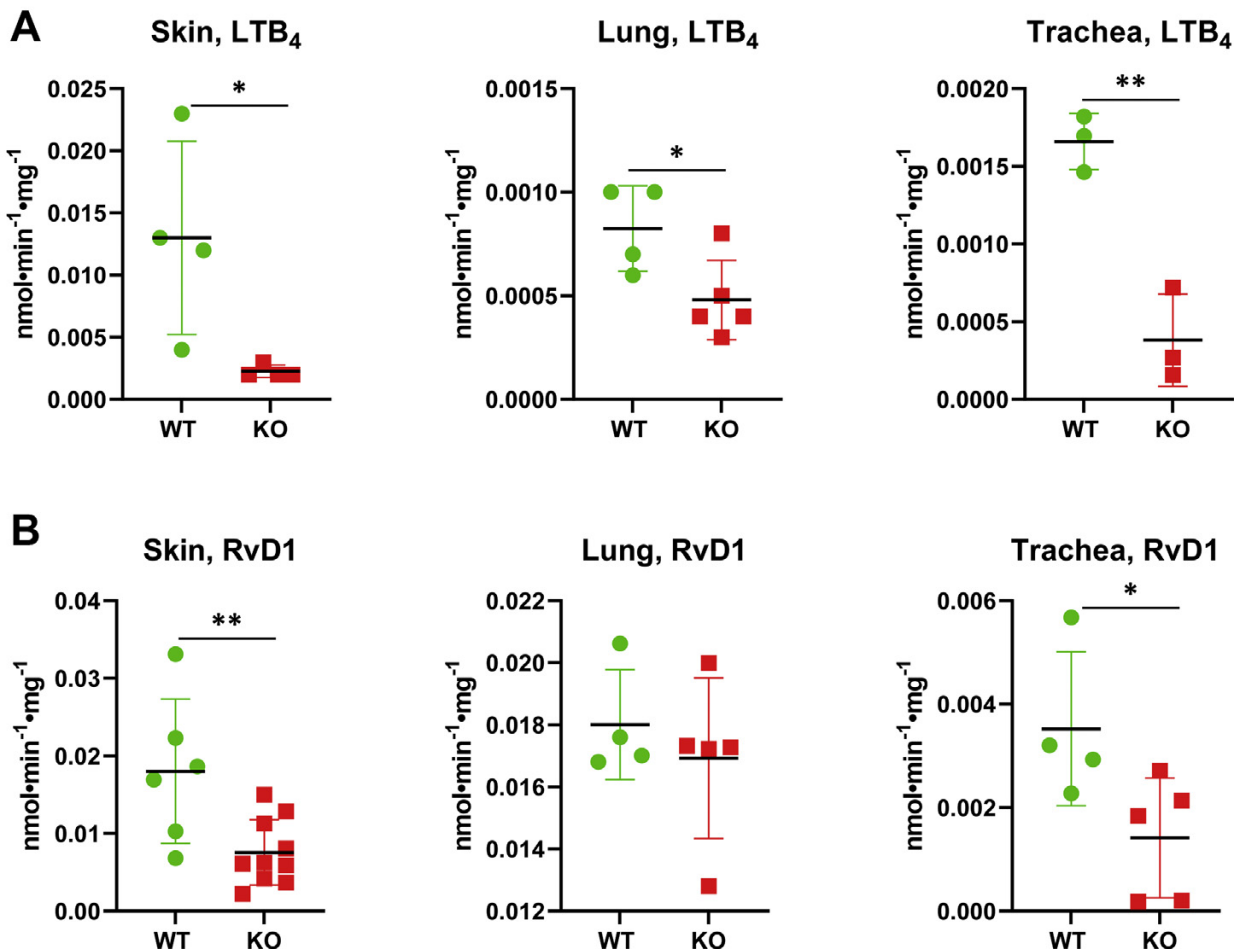
**Analysis of LTB4 and RvD1 dehydrogenase activity of microsomes isolated from wild-type (WT) *versus Dhrs9* gene knockout (KO) mice.** Microsomes (50 μg) of female mice (28–35 days old) isolated from the skin, lungs, and several tracheas pooled into three samples were incubated with 1 μM LTB_4_ (*A*) or 1 μM RvD1 (*B*) and 1 mM NAD^+^ for 1 h at 37 °C. The reaction products were analyzed by reversed-phase HPLC. The results are presented as mean ± S.D., ∗*p* ≤ 0.05; ∗∗*p* ≤ 0.01.

Previous studies suggested that the DHRS9 is highly expressed in the skin, lungs, and trachea (16, 23). In order to determine whether the absence of DHRS9 reduces the activities of microsomes from these tissues toward oxylipins, we first tested the microsomes from wild-type mice with a panel of oxylipins to identify the substrates that are metabolized by microsomes at a measurable rate. Significant activity was detected toward LTB_4_ with microsomes from the skin; whereas the activities of lung and tracheal microsomes were by an order of magnitude lower (Fig. 5*A*). A comparison of microsomal activities of the skin from wild-type littermates *versus Dhrs9*^*−/−*^ mice showed a sixfold reduction in the rate of metabolic conversion of 1 μM LTB_4_ with DHRS9-null microsomes. Tracheal microsomes displayed a fourfold decrease, and lung microsomes—a 1.7-fold decrease in the rate of LTB_4_ oxidation compared with corresponding microsomes from the wild-type mice (Fig. 5).

A measurable oxidation rate by the skin and lung microsomes was also detected with 1 μM RvD1, whereas the rate with tracheal microsomes was about tenfold lower (Fig. 5*B*). DHRS9-null mice displayed significantly reduced activities toward 1 μM RvD1 for microsomes from the skin (2.4-fold) and trachea (2.5-fold). Together with *in vitro* assays showing that LTB_4_ and RvD1 are good substrates for the recombinant mouse DHRS9, the comparison of microsomal activities of DHRS9-null mice with the wild-type indicated that the extent of DHRS9 contribution to the oxidative activity of microsomal membranes toward LTB_4_ and RvD1 varies across mouse tissues.

Notably, in agreement with the lower activities of recombinant DHRS9 toward allopregnanolone and all-*trans*-retinol, there were no differences in the oxidation rates of allopregnanolone or all-*trans*-retinol between the microsomes isolated from tissues of wild-type mice compared with *Dhrs9*^*−/−*^ mice (data not shown).

## Discussion

Most of the studies published to date refer to DHRS9 as a retinol dehydrogenase based on the previously published reports (16, 17, 18, 36). However, as shown in the present study, DHRS9 is much more efficient toward oxylipins with hydroxyl groups at positions 12 and above than toward retinol or androsterone. It also appears that the members of the SDR9C family have more divergent functions than the members of the SDR16C or SDR7C families, which primarily specialize in the oxidative (SDR16C) or reductive (SDR7C) metabolism of retinoids. Unlike human DHRS9, human RDH16 displayed much lower rates and higher *K*_m_ values toward oxylipins than toward androsterone. This study utilized the same Sf9 microsomal preparations of SDRs that were previously analyzed for their activity toward all-*trans*-retinol and 3α-hydroxysteroids as substrates (34). To determine whether there was a loss of activity during storage, the microsomes were retested with saturating concentrations of all-*trans*-retinol and androsterone. These assays demonstrated that no significant loss of activity had occurred; thus, the catalytic efficiency of SDRs toward C18-C22 polyunsaturated hydroxylipids can be compared with the previously published data on all-*trans*-retinol and androsterone.

Based on the previously reported parameters (34), the catalytic efficiency value (*V*_max_/*K*_m_) of microsomal preparations containing RDH16 toward androsterone was 24 pmol/(min × μM) and only ∼0.1 pmol/(min × μM) toward all-*trans*-retinol (Table 3). Thus, androsterone appeared to be a much better substrate for RDH16 than all-*trans*-retinol and a somewhat better substrate than oxylipins, although RDH16 could potentially metabolize these compounds if they were more abundant than androsterone. In comparison, human HSD17B6 appeared to display a strong preference for androsterone compared with any other substrates tested. Its *V*_max_/*K*_m_ value for androsterone was 150 pmol/(min × μM) (Table 4) (10).

Since rat RDH7 was one of the founding members of the SDR9C family (6), we also examined its activity toward oxylipin substrates. To our surprise, this enzyme displayed high catalytic efficiency for the oxidation of 15(*S*)-HETE and 9(*S*)-HODE (Table 5), suggesting that rat RDH7 could participate in the oxidative metabolism of these substrates. Thus, like human RDH16, which is expressed primarily in the liver (9), the liver-specific rat RDH7 (6) may also contribute to the oxidation of oxylipins in a tissue-specific manner.

It should be noted that mice have more members of the SDR9C family than humans and rats (Fig. 1) (4). Several of these proteins, *i.e.*, mouse RDH16, RDH7, and RDH9, were shown to exhibit catalytic activity toward 3α-hydroxysteroids and *cis*-retinols and were named *cis*-retinol/androgen dehydrogenases (CRAD) 1, 2, and 3, respectively (37, 38, 39). Whether these enzymes are also active toward oxylipins has not yet been reported; however, potentially, they could complement the activity of mouse DHRS9 in a tissue-specific manner.

This investigation identified human DHRS9 as the most potent oxylipin dehydrogenase among the tested human members of the SDR9C family. Importantly, the ability of DHRS9 to metabolize oxylipins seems to be conserved in mice, supporting the physiological relevance of DHRS9 in the metabolism of these compounds. Furthermore, activity assays using microsomes from mouse tissues show that the microsomal activity toward LTB_4_ and RvD1 is significantly decreased in the skin and trachea of DHRS9-null mice compared with wild-type mice, providing further evidence in support of DHRS9 role in metabolism of oxylipins.

Previous studies suggested that cytosolic NAD^+^-dependent SDR36C1, 15-hydroxyprostaglandin dehydrogenase (15-PGDH), which is the primary enzyme involved in metabolism of prostaglandins (40), also displays activity toward lipoxins and other 15-hydroxy lipoxygenase products (41, 42, 43). However, based on the published values for *V*_max_ (18,083 nmol/(min × mg)) and *K*_m_ (16 μM) (41), the catalytic efficiency 15-PGDH with 15(*S*)-HETE was 1130 pmol/(min × μM) when calculated based on the activity in samples containing 1 μg of purified 15-PGDH. In comparison, the *V*_max_ value of partially purified human DHRS9 in Sf9 microsomes for 15(*S*)-HETE was 390 pmol/(min × μM). Based on our estimates, DHRS9 protein in this preparation constitutes 1/10th of the microsomal protein; hence, its catalytic efficiency for 15(*S*)-HETE is higher (∼3900) than that of 15-PGDH. The membrane-bound DHRS9 might have an additional advantage over the cytosolic 15-PGDH in terms of its access to lipophilic substrates.

In fact, one of the intriguing possibilities with respect to the physiological role of DHRS9 might be its participation in the so-called 12-hydroxyeicosanoid dehydrogenase/10,11-reductase pathway, which was implicated in metabolism of 12(*S*)-HETE, LTB_4_, and possibly of 13(*S*)-HODE (44, 45). Pioneering studies carried out by Wainwright and Powell demonstrated that catabolic conversion of 12(*S*)-HETE and LTB_4_ is initiated when both compounds are rapidly oxidized by 12-hydroxyeicosanoid dehydrogenase followed by reduction of the 10,11-double bond by 10,11-reductase (44). Of prime significance, their biochemical characterization of 12-hydroxyeicosanoid dehydrogenase revealed that this is a microsomal NAD^+^-dependent enzyme (44). Around this time in the 1990s, a cytosolic NADP^+^-dependent LTB_4_ 12-hydroxydehydrogenase was purified and cloned by Yokomizo *et al.* (46, 47). It turns out that this enzyme, one of the zinc-independent members of the medium-chain dehydrogenase/reductase (MDR) superfamily, is identical to prostaglandin 13-reductase (48). In view of the 10:1 relative abundance of NADPH over NADP^+^ in cells, it is likely that its reductive functions predominate, and this is further suggested by recent direct analyses indicating 50-fold more efficient activity as a prostaglandin Δ13-reductase compared with its oxidation of LTB_4_ (49).

To our knowledge, to date, the molecular nature of the microsomal NAD^+^-dependent 12-hydroxyeicosanoid dehydrogenase is not established. Based on the kinetic analysis reported here, it seems reasonable to propose that the 12-hydroxyeicosanoid dehydrogenase activity described by Wainwright and Powell might reflect the activity of microsomal DHRS9. If this is the case, it suggests that DHRS9 plays a pivotal role in metabolism of LTB_4_, which is a potent proinflammatory agent and, potentially, in metabolism of other oxylipins that had been identified as substrates of DHRS9 in this study. Certainly, as established by Yokomizo *et al.*, (46) the oxidation of LTB_4_ to 12-oxo-LTB_4_ results in 100-fold loss of bioactivity, indicating that the reaction catalyzed by 12-hydroxyeicosanoid dehydrogenase can efficiently terminate LTB_4_ signaling.

The expression levels of DHRS9 in human and rodent tissues vary greatly depending on the underlying conditions. DHRS9 gene is frequently cited as a differentially expressed gene in such diseases as colorectal cancer (29), cicatricial alopecia (30), polycystic ovary syndrome (31), oral squamous cell carcinoma (32), rheumatoid arthritis (50), pancreatic cancer (51), *etc*. Consequently, examination of the physiological role of DHRS9 in the metabolism of oxylipins initiated with this study should shed light on the contribution of DHRS9 to pathophysiology of cancer progression and potentially other diseases linked to inflammation.

## Experimental procedures

### Reagents

Oxylipins were purchased from Cayman Chemical. Retinoids were purchased from Toronto Research Chemicals and steroids from Steraloids and Sigma.

### Expression vectors

Constructs for expression of human DHRS9, RDH16, HSD17B6, RDH5, RDH10, HSD11B1, in Baculovirus system, as well as HEK293 cell line stably expressing human DHRS9 were described previously (9, 10, 15, 21, 23). Vector containing cDNA encoding rat RDH7 was provided by Dr Gregg Duester at Sanford Burnham Prebys Medical Discovery Institute. For expression in Sf9 cells, RDH7 cDNA was subcloned in EcoRI–NotI sites of pVL1393 Baculovirus transfer vector.

Coding sequences of mouse and rat DHRS9 including the stop codons were amplified from the skin and heart cDNA, respectively, and cloned into BamHI – EcoRI sites of pCMV-Tag4a vector (Stratagene) without tags. The primers used for preparation of constructs are listed in Table S2.

### Determination of kinetic constants

SDR proteins were expressed in Sf9 cells using the Baculovirus expression system as described previously and microsomal preparations containing the recombinant proteins were used for activity assays (9, 10, 15, 35). Steady-state kinetics were performed in 90 mM potassium phosphate, pH 7.3, and 40 mm KCl at 37 °C in siliconized glass tubes. Oxylipin substrates were added from 100× stocks prepared in ethanol with final concentration of ethanol in reactions 1%. The 500-μl reactions were started with the addition of cofactor and stopped by addition of 2.5 ml of methylene chloride (LTB4, RvD1, 17(*R*)-RvD1, RvE1, LXA4, LXAB4), or 0.5 ml of ethyl acetate and 2 ml of hexane (12(*S*)-HETE, 15(*S*)-HETE, 5(*S*)-HETE, 9(*S*)-HODE, 13(*S*)-HODE, 14(*S*)-HDoHE). The aqueous phase was acidified to pH ∼3.0 by the addition of 10 μl of 4N HCl to allow for the extraction of carboxylic acids. Samples were vortexed and centrifuged in a low-speed benchtop centrifuge to facilitate phase separation. Organic phases were collected, and solvent was evaporated under flow of N_2_. Dry residues were dissolved in 100 μl of the mobile phase solvent, and 50 μl of the samples was analyzed using reverse-phase HPLC. The HPLC system consisted of Waters e2695 separation module, Waters 2998 photodiode array detector, and Waters Symmetry C18 column, 4.4 × 150 mm, 3.5 μM (Waters). Separation was performed in the isocratic mode at 0.7 ml/min flow rate with 50:50 acetonitrile:water mobile phase for LTB_4_, RvD1, 17(*R*)-RvD1, RvE1, LXA_4_, LXB_4_, and with 80:20 acetonitrile:water mobile phase for the remaining compounds. Mobile phase also contained 0.01% of glacial acetic acid.

Chromatograms were extracted at maximum absorbance wavelength for each of the analyzed compounds. The under-the-curve areas of the substrate and product peaks were converted into molar amounts using linear regressions of the calibration curve obtained by the triplicate injections of four serial dilutions of each compound (covering range of 10–1250 pmol per injection) from the commercial stocks with known concentrations. Steady-state kinetics were performed in 90 mM potassium phosphate, pH 7.3, and 40 mm KCl at 37 °C in siliconized glass tubes using microsomal membranes containing individual SDR enzymes as described previously (9).

For determination of apparent *K*_m_ values, six concentrations between 0.25 and 16 μM were used for 12(*S*)-HETE and 13(*S*)-HODE; 0.125 to 16 μM for 15(*S*)-HETE and 9(*S*)-HODE; 0.125 to 1.25 μM for 14(*S*)-HDoHE; 0.125 to 4 μM for LTB_4_ and RvD1; and 0.125 to 8 μM for LXA_4_. Initial velocities (nmol of product formed/mg of microsomal protein) were obtained by linear regression. The amount of product formed was less than 15% within the 15-min reaction time and was linearly proportional to the amount of microsomes added. Each *K*_m_ determination was repeated at least three times. A control without added cofactor was included with each experiment.

Reference activity with ADT was determined as described in reference (10) using 5 μg of Sf9 microsomes containing the respective enzymes, which were incubated with 20 μM ADT and 1 mM NAD^+^ for 15 min in 500-μl reaction volume at 37 °C (Fig. S2). The radiolabeled 5α-androstan-3α-ol-17one (androsterone) (45 Ci/mmol) (NEN Life Science Products) was diluted with cold steroid (Sigma) to achieve the required specific radioactivity. Reference activities for all-*trans*-retinol were determined as described in (35) under the same conditions as for ADT except the concentration of all-*trans*-retinol was 10 μM.

### Western blot analysis

Protein concentrations were determined using BioRad DC Protein Assay with BSA as a standard. Total protein homogenates or microsomal protein fraction as indicated was subjected to SDS-PAGE in 12% separating gel with 4% stacking gel. The gels were transferred to Immobilon-P PVDF membrane (Millipore) and blocked in 4% BSA in TBST buffer.

Blots were incubated overnight at 4 °C in blocking buffer with the following primary antibodies: DHRS9 rabbit polyclonal antibody (#A6324, Abclonal) at 1:2000 dilution, cytochrome P450 reductase antibodies (#AB1257, Chemicon International) at 1:4000 dilution or with custom-made rabbit polyclonal DHRS9 antibodies (1:250), and cytochrome p450 antibodies ab #13513 (Abcam, 1:1000). Secondary goat anti-rabbit horseradish-peroxidase-conjugated antibody (#170-6515, Bio-Rad) was used at 1:10,000 dilution. Immunoreactive bands were visualized using the Pierce ECL Western Blotting Substrate (#32106, Thermo Scientific). Alternatively, ECL Plex goat anti-rabbit Cy5 antibody (#GEPA45011, Sigma) was used at a 1:2500 dilution. Imaging was performed using ChemiDoc MP Imaging System (Bio-Rad). Blots were quantified with UN-SCAN-IT software (Silk Scientific Inc).

### Generation of *Dhrs9*^*−/−*^ mice

The studies were approved by the UAB IACUC Board. Mouse C57BL/6NTac ES cells carrying targeted knockout allele *Dhrs9*^*tm1(KOMP)Vlcg*^ were obtained from KOMP Repository at the University of California–Davis. The insertion of ZEN-UB1 targeting cassette in *Dhrs9*^*tm1(KOMP)Vlcg*^ allele (Velocigene KOMP Definitive Null Allele Design #13240, http://velocigene.com/komp/detail/13240) leads to the deletion of 8577 bp on mouse chromosome 2 corresponding to 69,223,257 to 69,231,833 nt in the genome build GRCm39. The deleted fragment includes exons 2 to 5 of the reference transcript NM_175512 and eliminates an entire protein coding sequence of *Dhrs9*. The inserted 6084-bp cassette encodes a LacZ reporter expressed under the control of endogenous *Dhrs9* promoter and neomycin selection marker under the control of human ubiquitin C promoter.

Chimeric animals were generated by the University of Alabama at Birmingham Transgenic and Genetically Engineered Models facility *via* ESC injection into albino C57BL/6J blastocysts. Male chimeras were crossed to wild-type C57BL6/J females to establish germline transmission. Heterozygous founders were identified by PCR with DHRS9-WTF (5′-GAT GGA TGA GTT TGC GTG TG-3′) and DHRS9-WTR (5′-TCA GGA CCT TTA TGC CTG TTC-3′) primers for wild-type allele (942 bp product), and DHRS9-WTF and (5′-GTC TGT CCT AGC TTC CTC ACT G-3′) primers—for knockout allele (504 bp product). The same primers were used for subsequent genotyping of progeny obtained from crossing of heterozygotes.

### Statistical analysis

Statistical significance was determined using two-tailed unpaired *t* test.

## Data availability

All data are contained within the manuscript.

## Supporting information

This article contains supporting information (10).

## Conflict of interest

The authors declare that they have no conflicts of interest with the contents of this article.
