# Supplementary material for: Dehydrogenase reductase 9 (SDR9C4) and related homologs recognize a broad spectrum of lipid mediator oxylipins as substrates
Source: J Biol Chem. 2021 Dec 22;298(1):101527. doi: 10.1016/j.jbc.2021.101527 (PMC8761697; doi:10.1016/j.jbc.2021.101527)
Supplement: Supplemental Figures S1, S2, Tables S1 and S2 [file mmc1.pdf]

SUPPORTING INFORMATION

Dehydrogenase reductase 9 (SDR9C4) and related homologs recognize a broad spectrum of lipid mediator oxylipins as substrates

Olga V. Belyaeva,[#] Samuel E. Wirth,[#] William E. Boeglin,[Δ] Suman Karki,[#] Kelli R. Goggans,[#] Stacy G. Wendell,[&] Kirill M. Popov,[#] Alan R. Brash,[Δ] and Natalia Y. Kedishvili[#]*

Table of Contents

Table S1. NCBI nomenclature of human and rodent genes from the SDR9C protein family.

Table S2. Primers used for preparation of constructs.

Figure S1. Structures of substrates used in the present study.

Figure S2. Activity of SDR9C enzymes toward 3α-hydroxyl group on androsterone.

Table S1. NCBI nomenclature of human and rodent genes from the SDR9C protein family.

Species	Gene name	Protein names
**human**	*DHRS9*	SDR9C4, DHRS9, hRDH-E2, RDHL
**mouse**	*Dhrs9*	DHRS9
**rat**	*Dhrs9*	DHRS9, eRolDH
**human**	*RDH16*	SDR9C8, RDH16, RoDH4, RDHE
**rat**	*Rdh7*	RDH7, RoDH1, Rdh3
**human**	*HSD17B6*	SDR9C6, HSD17B6, RoDH, RL-HSD, HSE
**rat**	*Hsd17b6*	HSD17B6, HSD17B9

Table S2. Primers used for preparation of constructs.

Construct	Vector	Cloning site	Method	Primer name	Primer sequence
Mouse DHRS9 untagged	pCMV-Tag4a	BamHI-EcoI	amplified from mouse skin cDNA	mDhrs9BamF	AAA GGA TCC atg ctg ttt tgg ttg ttg gct
				mDhrs9EcoR	A AAG AAT tca cac agc ttt ggg att tgc
Rat DHRS9 untagged	pCMV-Tag4a	BamHI-EcoI	amplified from rat heart cDNA	rDHRS9BamF	TTT GGATCC ATGCTGCTTTGGGTGTTGGCC
				rDHRS9EcoR	TTT GAATTC TCACACAGCTTGGGGATTGGC
Rat RDH7 (RODH1)	pVL1393	EcoRI-NotI	subcloned with partial 5' and 3'UTR from another vector		No primers

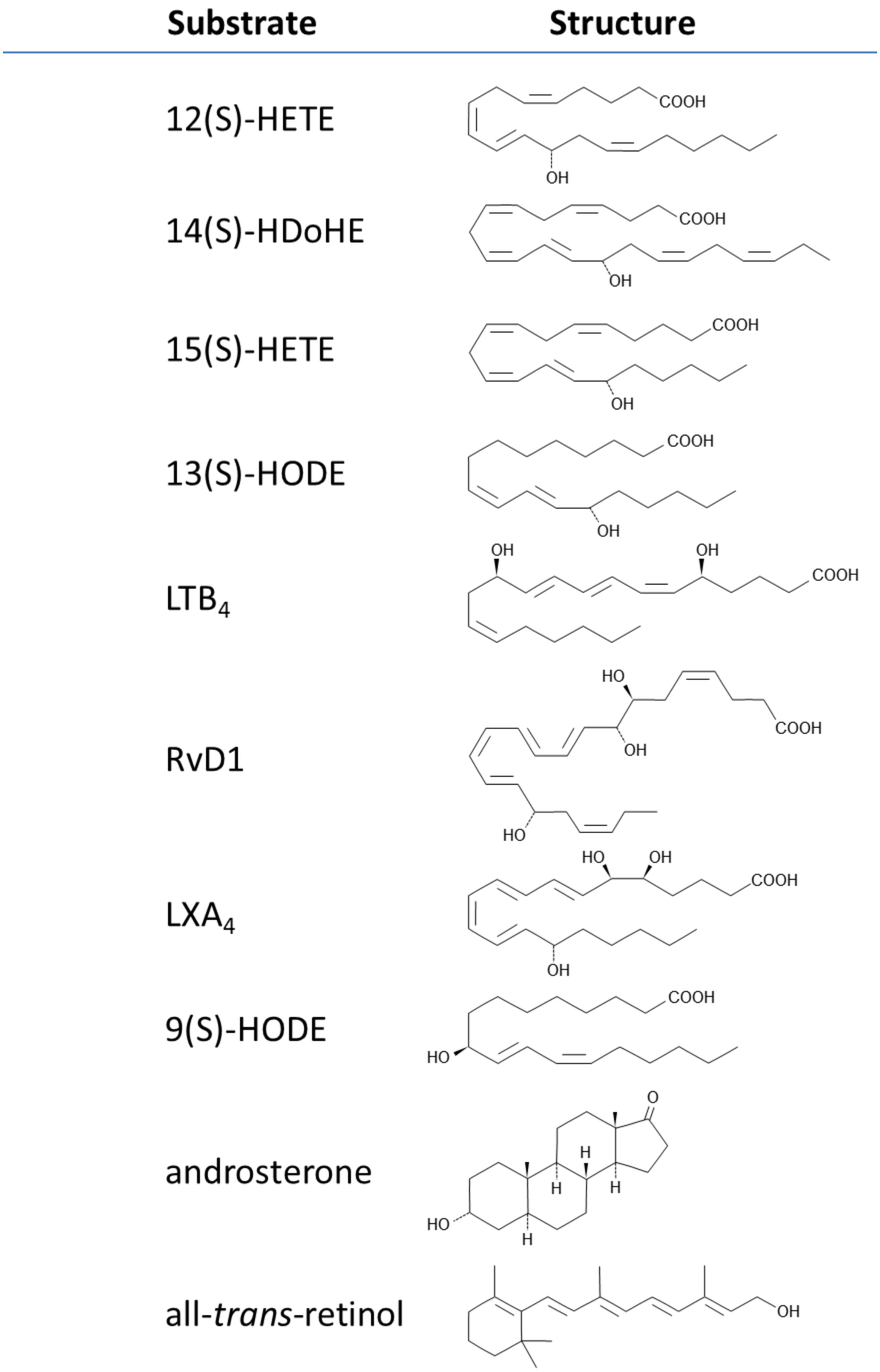

Figure S1. Structures of substrates used in the present study.

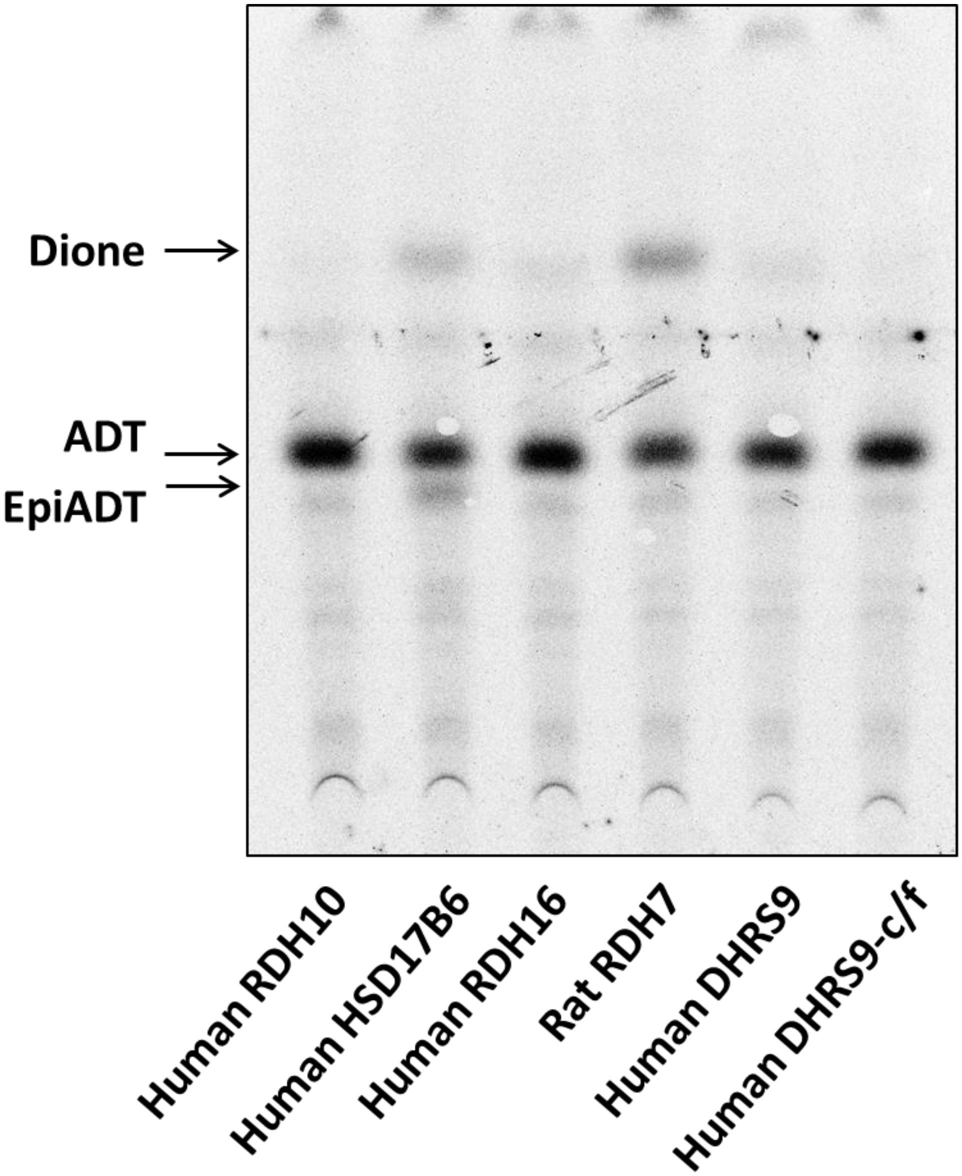

Figure S2. Activity of SDR9C enzymes toward 3α-hydroxyl group on androsterone. Sf9 microsomes (5 µg) containing the corresponding SDR proteins were incubated with androsterone (20 µM) for 15 min at 37 ºC. The reaction products were extracted and separated by thin-layer chromatography as described previously [10]. All four SDR9C proteins produced androstanedione (Dione) from androsterone (ADT). Note the appearance of the band corresponding to epi-androsterone (EpiADT) right below ADT in the lane containing human HSD17B6 protein, consistent with previous reports [10]. Human RDH10 Sf9 microsomes were included as a negative control. The last lane (Human DHRS9-c/f) shows the background activity of Sf9 microsomes without NAD^+. The image was obtained using Amersham Typhoon 5, GE Healthcare Bio-Sciences AB.
